# Low-dose decitabine increases peripheral natural killer T (NKT)-like cell proportions in patients with chronic myeloid neoplasms

**DOI:** 10.1016/j.cpt.2026.01.002

**Published:** 2026-01-21

**Authors:** Zhanqiang Zhang, Yue Wang, Haojun Zhang, Jinjing Zhao, Zhili Yang, Xiaoqian Wang, Yujie Tang, Xuechun Lu, Rui Xu

**Affiliations:** a Department of Hematology, Beijing United Family Hospital, Beijing 100015, China; b National Institutes for Food and Drug Control, Beijing 102629, China; c School of Management, Shanxi Medical University, Taiyuan, Shanxi 030000, China; d Department of Hematology, PLA 305 Hospital, Beijing 100013, China; e Department of Hematology, The Second Medical Center & National Clinical Research Center for Geriatric Diseases, Chinese PLA General Hospital, Beijing 100853, China

**Keywords:** Low-dose decitabine, Myelodysplastic syndromes, Primary myelofibrosis, Myelomonocytic, Natural killer T-cells

## Abstract

Decitabine is widely used in the treatment of chronic myeloid neoplasms, potentially through its immunomodulatory effects on CD8^+^ T cells and natural killer (NK) cells. However, as decitabine is often administered in combination with other agents and at varying dosages, the specific effects of low-dose decitabine alone remain unclear. This study aimed to investigate whether low-dose decitabine alone influences immune cell populations. Twelve patients with chronic myeloid neoplasms, including eight with myelodysplastic syndrome, three with myelofibrosis, and one with chronic myelomonocytic leukemia (CMML), received intravenous decitabine at 5 mg/m^2^ for seven days per 28-day treatment cycle. Peripheral blood samples were collected before and after decitabine treatment to assess immune cell proportions and their potential correlation with clinical response. No significant differences were observed in natural killer cells, T cells, CD8^+^ T cells, CD4^+^ T cells, or regulatory T cells (Tregs) following decitabine treatment. However, the proportion of NKT-like cells significantly increased from 3.5% to 4.25% (*P* = 0.035). Among the three patients who received at least three cycles of decitabine, improvements in anemia and thrombocytopenia were observed in those with elevated NKT-like cell levels. These findings suggest that low-dose decitabine may enhance the NKT-like cell population, which may be associated with therapeutic responses in chronic myeloid neoplasms.

## Highlights

• Low-dose decitabine (5 mg/m^2^) alone was administered for seven days in each 28-day treatment cycle to treat chronic myeloid neoplasms.
• Decitabine significantly increased the proportion of natural killer T (NKT)-like cells, but not that of NK cells and T cells.
• Among the three patients who received at least three cycles of decitabine, improvements in anemia and thrombocytopenia were observed in those with elevated NKT-like cell levels.

## Introduction

Decitabine (DAC) is a demethylating agent which is widely used for the treatment of chronic myeloid neoplasms, including myelofibrosis, myelodysplastic syndrome, and chronic myelomonocytic leukemia.^1^ One proposed mechanism of its therapeutic effects involves the promotion of CD8^+^ cytotoxic T cells^2^ and natural killer (NK) cells.^3^ However, most studies have investigated DAC in combination with other agents, such as venetoclax and granulocyte colony-stimulating factor,^4^ making it difficult to determine whether DAC alone is responsible for increasing CD8^+^ T cells and NK cells. Additionally, different doses of DAC exhibit distinct immunomodulatory effects.^5^ To clarify whether low-dose DAC can increase CD8^+^ T cell and NK cell frequencies, we treated a cohort of patients with low-dose DAC monotherapy and observed that it did not increase CD8^+^ T cells and NK cells, but instead led to an increase in NKT-like cells.

## Case report

Patients diagnosed with chronic myeloid neoplasms and deemed ineligible for intensive chemotherapy were included. Diagnoses of myelodysplastic syndrome, myelofibrosis, and chronic myelomonocytic leukemia were confirmed based on bone marrow morphology, immunophenotyping, cytogenetics, and molecular genetics. Inclusion criteria included an Eastern Cooperative Oncology Group performance status 0 or 1 and a life expectancy of ≥ 3 months. DAC was administered intravenously at a dose of 5 mg/m^2^ for 7 consecutive days in a 28-day treatment cycle until study withdrawal due to disease progression, unacceptable toxicity, or other reasons. Clinical responses and bone marrow remission status were assessed after at least two treatment cycles, following the international working group's recommended criteria. Overall survival (OS), progression-free survival (PFS), and event-free survival (EFS) were evaluated. Events included failure to complete at least two treatment cycles. Peripheral blood samples were collected on day 0 (pre-DAC) and day 8 (post-DAC) of each cycle of DAC treatment for flow cytometry analysis of T cell, natural killer (NK) cell, and NKT-like cell proportion. The study was approved by the Institutional Review Board of PLA 305 Hospital, and written informed consent was obtained from all participants.

Peripheral blood mononuclear cells were isolated using Ficoll-Hypaque gradient centrifugation (Amersham Biosciences, Piscataway, NJ, USA). Cells were incubated with fixable viability dye eFluorTM 506 (eBioscience, San Diego, CA, USA) in phosphate-buffered saline at 4 °C for 20 min, followed by incubation with fluorophore-conjugated monoclonal antibodies at 4 °C for 30 min, according to the manufacturer's instructions. NKT-like cells were defined as CD3^+^CD56^+^CD8^-^, while T cells, CD4^+^ T cells, CD8^+^ T cells, Tregs and NK cells were characterized as CD3^+^CD56^-^, CD4^+^CD3^+^CD56^-^, CD8^+^CD3^+^CD56^-^, CD4^+^CD25^+^Foxp3^+^, and CD3^–^CD56^+^, respectively. Flow cytometric analysis was performed using a Gallios Flow Cytometer with Kaluza Analysis Software (Beckman Coulter, Brea, CA, USA).

Data are presented as the mean ± standard deviation. Normality was assessed using the Shapiro-Wilk test. For normally distributed paired data (pre- and post-treatment), comparisons were made using paired *t*-tests. Survival analysis was conducted using the Kaplan-Meier method. Some lost-to-follow-up patients could not be tracked, so intention-to-treat (ITT) analysis was adopted, with lost-to-follow-up patients regarded as treatment failures. Statistical analysis was performed using SPSS version 18.0 (IBM Corp., Armonk, NY, USA). A *P*-value <0.05 was considered statistically significant.

Between February 2016 and August 2017, 12 patients enrolled (seven men and five women, median age 63 years, range: 16–81 years). The diagnoses included myelodysplastic syndrome (*n* = 8), myelofibrosis (*n* = 3), and chronic myelomonocytic leukemia (*n* = 1). Only three patients completed at least three cycles of DAC treatment. Six patients failed to continue DAC treatment due to thrombocytopenia and neutropenia. One patient stopped DAC treatment because of heart failure, and one patient underwent haploidentical hematopoietic stem cell transplantation after two cycles of DAC treatment. In brief, six patients received one cycle of DAC, three received two cycles, two received three cycles, and one received four cycles, totaling 22 treatment cycles. Low-dose DAC treatment was well-tolerated; only one patient developed heart failure, which resolved following digoxin therapy. None of the twelve patients developed leukemic transformation.

**Figure 1:**
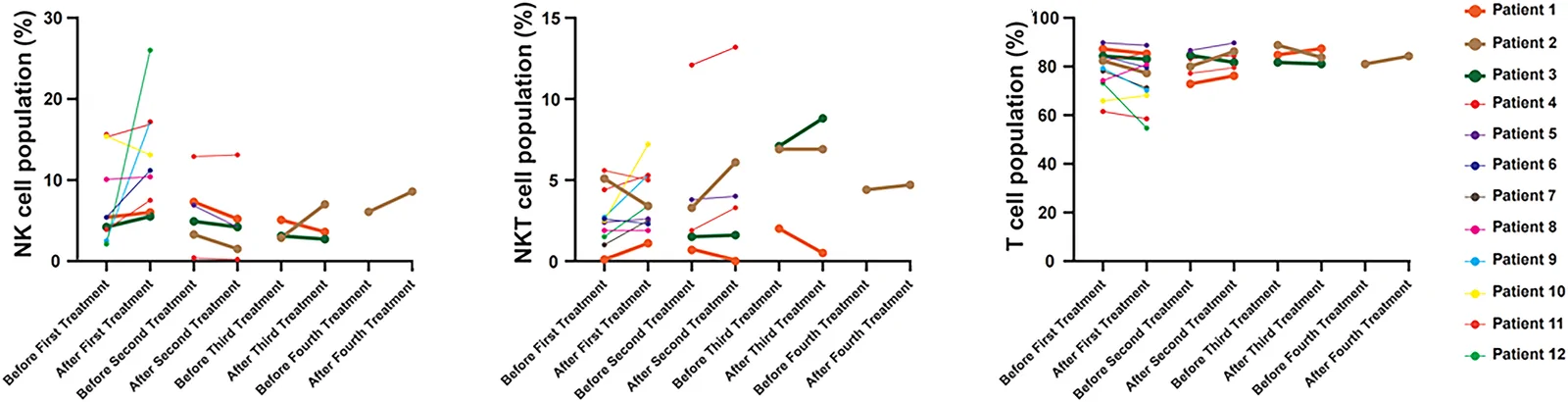
Dynamics of NK, NKT, and T cell populations in peripheral blood during low-dose decitabine treatment. Dynamic changes in the frequency of peripheral blood NK cells, NKT cells, and T cell subsets in 12 patients with MDS, PMF, or CMML during all cycles of low-dose decitabine treatment. Each line represents an individual patient. Lymphocyte subsets were analyzed via flow cytometry at different time points (before treatment and after each cycle). CMML: Chronic myelomonocytic leukemia; MDS: Myelodysplastic syndrome; NK: Natural killer; PMF: Primary myelofibrosis.

No significant changes were observed in T cells (80.1% vs 79.9%, *P* = 0.341), CD4^+^ T cells (42.7% vs 42.2%, *P* = 0.684), CD8^+^ T cells (32.6% vs 32.3%, *P* = 0.789), NK cells (6.2% vs 8.7%, *P* = 0.116), or Tregs (7.6% vs 7.6%, *P* = 0.961) from pre-to post-treatment. However, NKT-like cells increased significantly from 3.5% pre-treatment to 4.25% post-treatment (*P* = 0.035) [Figure 1]. The proportion of NKT-like cells increased by 0.75% from pre-treatment to post-treatment in high-risk MDS patients and 1.35% in low-risk MDS patients. There was no significant difference between the two risk groups (*P* = 0.662).

Clinical responses were evaluated in three patients who completed at least three cycles of DAC.

• Patient 1 (CMML) exhibited an initial increase in NKT-like cells after the first cycle, followed by a decrease in subsequent cycles, accompanied by a decline in hemoglobin levels.
• Patient 2 (myelofibrosis) showed no initial NKT-like cell increase after the first cycle, but demonstrated a progressive increase from the second to fourth cycle, coinciding with anemia improvement.
• Patient 3 (myelodysplastic syndrome) received three cycles of DAC and exhibited a continuous increase in NKT-like cells. This patient's thrombocytopenia improved, with platelet counts normalizing post-treatment [Table 1 and Figure 1].

**Table 1:** Baseline characteristics and treatment response of patients treated with low-dose DAC.

Case	Age (years)/Sex	Diagnosis	Cytogenetic abnormalities	Gene mutations	Risk stratification (IPSS-R/DIPSS)	Comorbidities	Previous treatment	DAC cycle	HB-BD /HB-AD (g/L)	PLT-BD/PLT-AD ( × 10^9^/L)	Bone marrow response to DAC	Outcome at last follow-up (months or years after DAC)
1.	63/M	CMML1	Normal karyotype	None	NA	Coronary artery disease	None	3	115/97	691/528	SD	Lost to follow-up
2.	44/M	PMF	NA	*CALR*	Intermediate-2	Gouty arthritis	None	4	49/82	327/384	SD	Death from heart failure (5.6 years)
3.	81/F	MDS-MLD	Normal karyotype	None	Low	Hypertension, diabetes	None	4	107/139	56/129	PR	Death from pneumonia (4 months)
4.	64/F	PMF	+1, der (1; 13) (q10; q10)	*JAK2 V617F*	Intermediate-2	Hepatitis B	Interferon, hydroxyurea	2	130/88	218/141	SD	Alive (8.6 years)
5.	34/M	MDS-EB1	Del (20q13.2)	None	Intermediate	None	Erythropoietin, danazol	2	64/75	126/138	SD	Alive (8.1 years)
6.	78/F	PMF	NA	*JAK2 V617F*	Intermediate-2	Diabetes	Interferon	2	86/97	48/44	SD	Death from gastrointestinal hemorrhage (2.5 years)
7^a^.	64/M	MDS-EB1	-Y, −5, del (6q), +8, −15, −17	NA	High	None	None	1	NA	NA	NA	Lost to follow-up
8^a^.	64/M	MDS-EB1	Normal karyotype	NA	High	None	Erythropoietin, thalidomide	1	NA	NA	NA	Death from pneumonia (4.4 years)
9^a^.	50/M	MDS-MLD	Normal karyotype	NA	Very low	None	Hydroxyurea	1	NA	NA	NA	Death from heart failure (9 months)
10^a^.	33/F	MDS-MLD	Normal karyotype	NA	Low	Cervical cancer	None	1	NA	NA	NA	Death from unknown cause (1.7 years)
11^a^.	63/F	MDS-MLD	Normal karyotype	NA	Intermediate	None	Danazol, prednisolone	1	NA	NA	NA	Death from unknown cause (4.4 years)
12^a^.	77/M	MDS-MLD	−21	NA	Intermediate	None	Cyclosporin, thalidomide	1	NA	NA	SD	Alive (8.5 years)

a Patients 7–12 did not complete at least two DAC treatment cycles; therefore, platelet counts before and after treatment were not assessed. CMML: Chronic myelomonocytic leukemia; DAC: Decitabine; DIPSS: Dynamic International Prognostic Scoring System for primary myelofibrosis; F: Female; HB-AD: Hemoglobin after DAC; HB-BD: Hemoglobin before DAC; IPSS-R: Revised International Prognostic Scoring System for myelodysplastic syndrome; M: Male; MDS: Myelodysplastic syndrome; MDS-EB1: MDS with excess blasts (blasts make up 5%–9% of the cells in the bone marrow, or 2%–4% of the cells in the blood); MDS-MLD: MDS with multilineage dysplasia; NA: Not available; PLT-AD: Platelet after DAC; PLT-BD, Platelet before DAC; PMF: Primary myelofibrosis; PR: Partial remission; SD: Stable disease.

Bone marrow remission status was assessed in six patients with at least two treatment cycles. The partial remission rate was 16.7% (1/6), and the stable disease rate was 83.3% (5/6). The median follow-up duration was 23.6 months (interquartile range: 20.5–24.5 months). The last follow-up date was October 31, 2024. At that time, three patients remained alive, while seven had died, and two were lost to follow-up. The median survival time was 4.4 years (95% confidence interval [CI]: 0.9–7.9 years). The median PFS was 4.4 years (95% CI: 0.9–7.9 years). The median EFS was 5.6 years (95% CI: 1.9–9.3 years). The estimated 3-year survival rate was 66.7% (95% CI: 44.7%–99.5%), and the 5-year survival rate was 50.0% (95% CI: 28.4%–88.0%). Based on whether NKT-like cells increased after the first DAC treatment, patients were divided into two groups: the NKT-like cell-increased group and the NKT-like cell-decreased group. The mean survival time was 6.5 years (95% CI: 4.0–9.0 years) and 4.2 years (95% CI: 3.0–5.5 years) for the increased and decreased groups, respectively. The log-rank test *P*-value was 0.095. Among the survivors, Patient 5 underwent haploidentical hematopoietic stem cell transplantation from his mother in 2018. Follow-up data were summarized in Table 1.

## Discussion

DAC is a key therapeutic agent for chronic myeloid neoplasms such as myelodysplastic syndrome and myeloproliferative diseases, and may help prevent disease recurrence post-allogeneic hematopoietic stem cell transplantation.^1^ Its immunomodulatory mechanism has been linked to increased CD8^+^ T cells and NK cells. However, our study found that low-dose DAC did not elevate CD8^+^ T cells or NK cells, but instead led to a significant increase in NKT-like cells.

NKT cells are a distinct lymphocyte subset that co-express T cell receptors (TCR) and natural killer cell receptors. NKT cells are divided into two main types: type I and type II. Type I NKT cells, also referred to as invariant NKT (iNKT), have a semi-invariant TCR consisting of *Va14-Ja18* and *Vβ* chains of limited diversity, are CD1d-restricted, and respond to the marine sponge-derived glycolipid α-galactosylceramide (α-GalCer) when bound to CD1d. Type II NKT cells are also CD1d-restricted but have diverse TCRs and do not react to α-GalCer, but instead react with other self-lipids such as sulfatide. The CD3^+^CD56^+^ cell population includes “true” CD1d-restricted NKT cells. Conventional T cells have also been reported to express CD56. Since it is unclear whether all CD3^+^CD56^+^ cells are CD1d-restricted, this population is often referred to as “NKT-like” cells.^6^ Identification of NKT cells by flow cytometry probe is technically challenging because we lack specific lipids-loaded-CD1d tetramers that reliably define invariant and diverse NKT cells. NKT cells are MHC class I-restricted without CD8 expression, being only CD4^+^ or CD4^−^CD8^-^.^7^ NKT-like cells phenotypically were further defined by CD3^+^CD56^+^CD8^-^ in our study, so NKT-like cells were mostly comprised of NKT cells.

The dosage of DAC is typically 20 mg/m^2^ in other studies,^3^ while in our study, it was administered at a dose of 5 mg/m^2^. Different doses have been shown to exert various effects on immune cells. For example, high-dose DAC can induce significant cytotoxicity in NK cells.^5^ Furthermore, in other DAC-related studies, blood samples are typically obtained at the time of adequate lymphocyte recovery or at the beginning of a later cycle.^2^ In the study, blood samples were collected on day 0 (before DAC treatment) and day 8 (after DAC treatment). Therefore, the observed changes in immune cells could reflect the immediate effect of DAC. The two reasons above may explain why we found that DAC increased NKT-like cells rather than NK cells or T cells. These findings suggest that low-dose DAC (5 mg/m^2^) may be better tolerated in older patients (≥ 65 years old) who cannot tolerate conventional doses or patients with comorbidities, and monitoring the proportion of NKT-like cells may provide additional information regarding therapeutic efficacy. If there is no increase in NKT-like cells after 2 treatment cycles, adjustments to the treatment regimen (such as dose escalation or combination with other drugs) should be considered.

Myeloid neoplasms, such as acute myeloid leukemia, express CD1d, so NKT-like cells can directly kill the malignant cells. Simultaneously, NKT-like cells can indirectly kill malignant cells by activating NK cells and dendritic cells.^8^ CD1 expression is suppressed in cancer patients, and demethylating agents such as azacitidine can upregulate CD1, promoting NKT cell expansion.^9^ This suggests that DAC may enhance NKT-like cell proliferation through a mechanism similar to azacitidine. In our study, three patients with increased NKT-like cells following DAC treatment demonstrated significant improvement in anemia and thrombocytopenia, suggesting a potential therapeutic role of NKT-like cells. Anemia and thrombocytopenia may be caused by the inhibition of malignant cells in myeloid neoplasms. As NKT-like cells can kill malignant cells, normal bone marrow hematopoiesis may recover.

The findings suggest that immunotherapy based on NKT-like cells may be useful for the treatment of chronic myeloid malignancies. However, the small sample size is a limitation, and future studies should identify NKT cells within NKT-like cell populations using lipids-loaded-CD1d tetramers. Furthermore, although an increase in the proportion of NK cells was observed in our study, the difference was not statistically significant, which may be owing to the small cohort size. Further studies are required to validate these findings.

## Authors contribution

Rui Xu and Xuechun Lu designed the study; Jinjing Zhao, Zhili Yang, and Xiaoqian Wang collected the data and performed the analyses; Yujie Tang and Haojun Zhang analyzed the data, which were presented in the figures. Zhanqiang Zhang and Yue Wang interpreted the data and wrote the manuscript; all authors had full access to the primary clinical trial data and had final responsibility for the decision to submit for publication.

## Ethics statement

All procedures followed were in accordance with the ethical standards of the responsible committee on human experimentation and with the *Helsinki Declaration* of 1975 (as last revised in 2000). The study was approved by the Institutional Review Board of PLA 305 Hospital (No. KYLL-SPJ-2024-6); written informed consent was obtained from all participants.

## Declaration of Generative AI and AI-assisted technologies in the writing process

The authors declare that generative artificial intelligence (AI) and AI assisted technologies were not used in the writing process or any other process during the preparation of this manuscript.

## Funding

None.

## Conflict of interest

The authors declare that they have no known competing financial interests or personal relationships that could have appeared to influence the work reported in this paper.
